# Phosphatidylinositol 3-kinase inhibitor(LY294002) induces apoptosis of human nasopharyngeal carcinoma in vitro and in vivo

**DOI:** 10.1186/1756-9966-29-34

**Published:** 2010-04-22

**Authors:** Hanguo Jiang, Desheng Fan, Gengyin Zhou, Xiaofang Li, Huihua Deng

**Affiliations:** 1 Department of Pathology, School of Medicine, Shandong University, 250012 Jinan, China; 2 Current Address: Department of Pathology, Guangdong Medical College, Zhanjiang, China; 3 Department of Pathology, Guangdong Medical College, 524023 Zhanjiang, China

## Abstract

**Background:**

To evaluate whether PI3K/Akt pathway could effect on apoptosis and its mechanism in nasopharyngeal carcinoma cells.

**Methods:**

The activation of the PI3K/Akt and its effect on CNE-2Z cells in vivo and in vitro was investigated by MTT assay, flow cytometry, western blot, ELISA, terminal deoxyribonucleotide transferase-mediated nick-end labeling assays (TUNEL), and immunohistochemical analyses, using PI3K inhibitor, LY294002.

**Results:**

The results showed that LY294002 inhibited the phosphorylating of Akt (S473), cell proliferation, and induced apoptosis in CNE-2Z cells. However, our experiment results also demonstrated that apoptosis-induced LY294002 was directly regulated by caspase-9 activation pathway.

**Conclusion:**

These data suggested that PI3K inhibitor, LY294002, induced apoptosis by caspase-9 activation pathway and might be as a potentially useful target for therapeutic intervention in nasopharyngeal carcinoma patients.

## Background

Nasopharyngeal carcinoma (NPC) has a distinct epidemiology and distribution, southern China and Southeast Asia are the highest risk areas, while rare in most parts of the world. Although many NPC patients may undergo radiation therapy for possibly cure and new strategies have improved survival for patients with metastasis, 30%-40% NPC patients die from local recurrence and metastasis. A better understanding of signaling pathways contributing to NPC survival and apoptosis will provide targets for new therapeutic agents.

The phosphatidylinositol-3 OH kinase (PI3K)/Akt signaling pathway has been shown to contribute to cancer survival, apoptosis, and regulating a variety of cellular processes. In particular, Akt serine/threonine kinase is believed to play a critical role in controlling the balance between cell survival and apoptosis [1]. Previous studies had shown that phosphatidylinositol 3,4,5-trisphosphate(PIP3) generated by PI3K acts as a lipid second messenger essential for the translocation of protein kinase B(Akt) to the plasma membrane [2,3]. Akt is phosphorylated at two sites, T308 in kinase domain and S473 in regulatory tail. Phosphorylation at T308 and S473 is essential for maximal Akt activation [2,3]. Phosphorylated Akt regulates the function of a broad array of intracellular proteins involve in fundamental processes including cell proliferation, cell death, cell motility/adhesion, cell transformation, neovascularization, and the inhibition of apoptosis [2-5]. PIP3 levels and Akt activation are regulated by the action of phosphatase and tensin homologue deleted from chromosome 10(PTEN). The Akt survival pathway is regulated negatively by PTEN lipid phosphatase, which selectively dephosphorylates the 3' site on polyphosphoiositides produced by PI3K [6,7]. Alterations of the PI3K/Akt pathway in human carcinomas have been reported [8-10]. Many studies demonstrated that PI3K/Akt pathway is constitutively activated in various cancers, including gastric, renal cell, ovarian, and lung cancers, and plays a critical role in tumor formation [9-12]. There is now convincing evidence that the alterations of the PI3K/Akt pathway is related not only to tumor progression but also to human resistance to radiation and systemic therapies.

LY294002 (2-4-morpholinyl-8-phenlchromone) is chemical inhibitor of PI3K, which has been used extensively to study the role of PI3K/Akt pathway in normal and transformed cells [13,14]. Inactivation of PI3K using LY294002 has been demonstrated to lead to the dephosphorylation of Akt at both T308 and S473, consequently inducing specific G1 arrest in cell growth and finally to cell apoptosis [15,16]. The inhibitors of PI3K also have antitumor activity *in vitro *and *in vivo *in a variety of tumor types [12,17-19], and it is possible that cells expressing constitutively active Akt become dependent on its survival-promoting effects. Although these results have been observed in many human cancers [18-20], the role of LY294002 in human nasopharyngeal carcinoma has not been well documented yet.

To evaluate the significance of Akt phosphorylation in proliferation and apoptosis of human nasopharyngeal carcinoma, we investigated the role of Akt phosphorylation and the effect of LY294002 in vitro and in vivo. Our goal was to confirm that the PI3K/Akt pathway might be a new therapeutic target on clinic treatment for nasopharyngeal carcinoma patients.

## Methods

### Cell culture

Human nasopharyngeal carcinoma cell line CNE-2Z was from Pathological Department of Guangdong Medical College. Cells were cultured in RPMI-1640 (Gibco, USA) supplemented with 10% fetal bovine serum(Gibco, USA), 1% penicillin-streptomycin (Life Technologies) at 37°C in a humidified incubator with a 5% CO_2 _atmosphere.

### Cell proliferation assay

The cells were seeded into 96-well plates(Corning, Lowell, MA, USA) at 5000 cells/well. Twenty-four hours after cells were seeded, the medium was removed and replaced in the presence of LY294002 (0 μmol/L, 10 μmol/L, 25 μmol/L, 50 μmol/L, and 75 μmol/L) dissolved in DMSO or DMSO only for an additional 24 h and 48 h. To avoid any nonspecific toxic effects of DMSO on cell growth, DMSO concentrations were maintained at 0.5% in all experiments. MTT dye (5 mg/mL, Sigma, Saint Louis, MO, USA) was added to each well. The reaction was stopped by the addition of DMSO(Sigma), and optical density was measured at 490 nm on a multiwell plate reader. Background absorbance of the medium in the absence of cells was subtracted. All samples were assayed in triplicate, and the mean for each experiment was calculated. Results were expressed as a percentage of control, which was considered to be 100%.

### Annexin V/PI for cell apoptotic analysis

Cells were collected with 0.25% trysin/0.02% EDTA after presence of LY294002(0 μmol/L, 10 μmol/L, 25 μmol/L, 50 μmol/L, and 75 μmol/L)24 h and 48 h. At the same time, caspase-9 specific inhibitor, ZVAD(0 μmol/L, 5 μmol/L, 10 μmol/L, 20 μmol/L), was added for 48 h. Cells were harvested at the end of treatment, rinsed twice with PBS, and stained with Annexin V-FITC apoptosis detection kit I (BD Biosciences). Analysis was performed on the FACS Calibur using CellQuest software.

### P-Akt ELISA assay

CNE-2Z cells were plated on 6-well plates in RPMI-1640 plus 10% FBS in duplicate for each treatment. Chemical inhibitor LY294002 was added to the appropriate wells. The cells were incubated at 37°C for 24 h and 48 h. Phosphorylated protein level of treated and untreated cells lysates was measured using a commercially available ELISA kit. Statistical analysis to determine significance of the observed differences was used by the Linear Regression. Experiments were repeated three times.

### Western blot analysis

Cells were homogenized in 500 μl with lysis buffer (1% Triton X-100, 50 mM Tris-HCL (Ph7.5), 0.1% SDS, 150 mM NaCl, 10% glycerol, 1.5 mM MgCl_2_, 1 mM PMSF, 0.1 mM Na V0_4_, 0.1 mM benzamidine, 5 μl/ml leupeptin, 5 μl/ml aprotinin). The lysates were clarified by centrifugation at 12000 g for 15 min at 4°C. Samples were analyzed by 15% SDS-polyacrylamide gels, and transferred to nitrocellulose membranes, and the membranes was incubated with primary antibodies, followed by horseradish peroxidase-cunjugated secondary antibodies. An antibody for β-actin was used as a loading control.

### Tumor xenograft experiments

All of the experiments involving animals in present study were approved by the animal center of Guangdong Medical College in accordance to institutional and Guangdong government guidelines for animal experiments. Athymic nude mice were used when they were 6-8 weeks. Mice were randomly divided into free separated into five groups (n = 4 mice). Mice were housed in the same environment with controlled temperature, humidity, and a 12 h light/dark cycle. Mice were inoculated subcutaneously with CNE-2Z cells (1 × 10^6 ^cells/mouse in 200 μl of RPMI-1640) into the flank. The tumor take rate was 100%. After 1 week, an intraperitoneal injection was performed to the xenograft mice with different dosage of LY294002 (10 mg/kg, 25 mg/kg, 50 mg/kg, and 75 mg/kg twice weekly (n = 4 mice), each group for 4 weeks. Treated mice were monitored any signs. Body weight and tumors size were measured twice a week. Tumor size was measured using calipers and tumor volume was calculated (volume = long axis × short axis^2^). At the end of the treatment, all mice were euthanized. One part of tumor tissue was fixed in formalin and embedded in paraffin, and another part was stored at -70°C.

### Immunohistochemistry analysis

Paraffin sections were used for immunohistochemical analysis of Akt, p-Akt, caspase-9, Ki67, and the TUNEL method for determining of DNA fragmentation. TUNEL assay was carried out according to the protocol of the ApopTag Peroxidase in situ apoptosis detection kit (Chemicon International, Temecula, Calif, USA). Positive expression of Akt, p-Akt, and caspase-9 locates in the cytoplasm. Immunohistochemical expression of Ki67 and TUNEL-positive cells shows in the nuclei. The mean percentage of positive tumor cells was determined from five areas at highpower field (×400). The growth index (GI) and the apoptosis index (AI) were calculated by counting the Ki67- and TUNEL-positive cells in a total of 1000 tumor cells observed from more than representative highpower fields, respectively. Immunohistochemical results were evaluated independently.

### Statistical analysis

Data were expressed as mean ± SD of mean and compared by unpaired Student's *t *test. ELISA Assay was used by the Linear Regression. Results were considered significant at a value of *P < 0.05*.

## Results

### Effects of PI3K/Akt inhibition on proliferation and apoptosis of NPC cells

**Figure 1:**
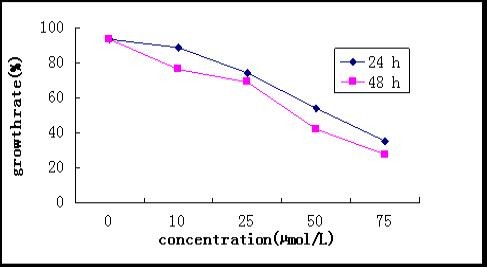
**CNE-2Z cells growth rate in different concentrations of LY294002**.

**Figure 2:**
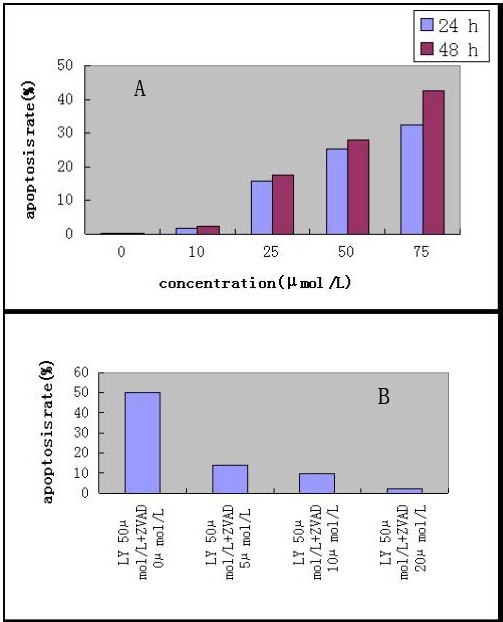
**CNE-2Z cells apoptosis rate**. CNE-2Z cells apoptosis rate induced by different concentrations of LY294002. B. CNE-2Z cells apoptosis inhibited by different concentrations of ZVAD (0, 5, 10, and 20 μmol/L) at 48 h.

To determine whether inhibition of PI3K/Akt activity(LY294002) would inhibit cell proliferation and promote apoptosis in CNE-2Z cell line, MTT assay and flow cytometry analysis were used. When the cells were cultured in medium containing different concentrations of LY294002 for 24 h and 48 h, cell proliferation was remarkably decreased in a dose-dependent fashion (Fig 1). The Annexin V/PI assay was used to detect apoptosis in NPC cells. As shown in Fig 2A, the proportion of apoptotic cells was significantly increased in dose-dependent. Caspase-9 is regarded as a most likely candidate relating with apoptosis-induced by PI3K inhibitor [21], we explored whether caspase-9 was involved in LY294002- induced cell apoptosis in CNE-2Z cells by detecting caspase-9 activity in cells treated with PI3K/Akt inhibitor. When caspase-9 specific inhibitor, ZVAD, was added, apoptosis rate was decreased at 48 h (Fig 2B).

### Effects of PI3K/Akt inhibition on Akt phosphorylation in NPC Cells

When LY294002 was added to NPC cells with different concentrations, levels of phosphorylation (S473) Akt were decreased in treated NPC cells, exhibiting a dose-response effect (Table 1).

**Table 1:** Expression of p-Akt protein in CNE-2Z cells treated with LY294002

LY294002 (mol/L)	n	P-Akt(unit/ml)
0	3	74.10 ± 1.00
10	3	62.65 ± 0.68
25	3	50.09 ± 1.83
50	3	25.22 ± 1.83
75	3	13.21 ± 1.34
F		1328.43
*P*		< 0.001

### Effects of PI3K/Akt inhibitionon protein expression in NPC cells

**Figure 3:**
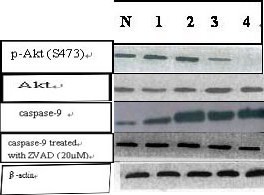
**Western blot analysis of Akt, phosphor-Akt(S473), caspase-9, and caspase-9 treated with ZVAD (20 μmol/L)**. N: no treatment group; Lanes1, 2, 3, and 4: treatment with LY294002(10 μmol/L, 25 μmol/L, 50 μmol/L, and 75 μmol/L respectively).

The results of Western blot showed that total Akt protein level was not difference with different concentration. In contrast, phosphorylated Akt (S473) expression levels were significantly decreased in treated group. At the same time, we explored whether caspase-9 was involved in LY294002- induced cell apoptosis in CNE-2Z cells by detecting caspase-9 activity in cells treated with PI3K/Akt inhibitor. The results show caspase-9 activity in CNE-2Z cells was up-regulated by LY294002, whereas the level of caspase-9 was not changed after using ZVAD (Fig 3).

### Effects of PI3K/Akt inhibition proliferation and apoptosis in vivo

**Figure 4:**
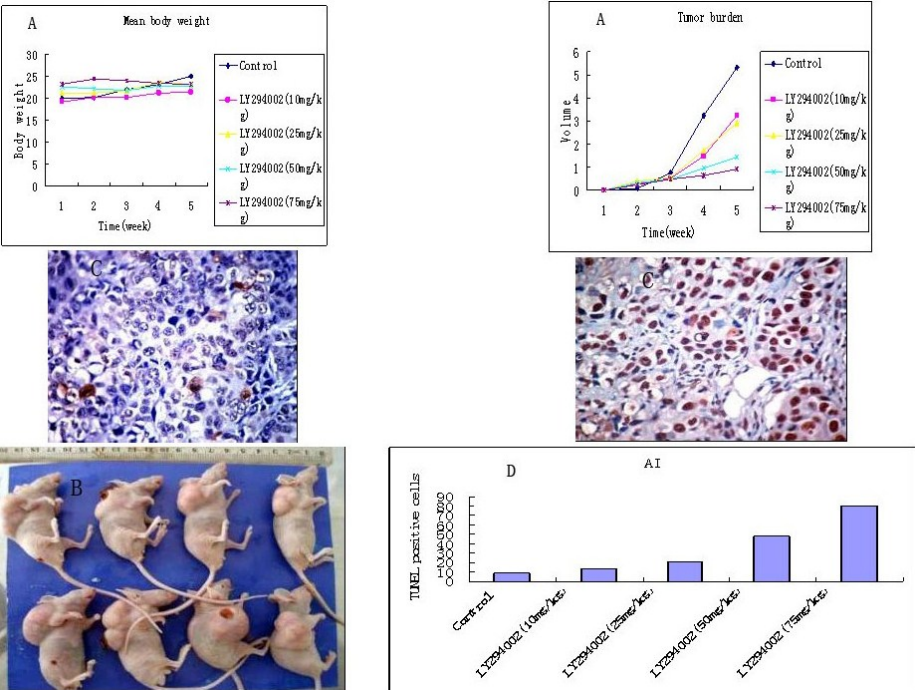
**Growth and apoptosis analysis of tumors xenografts in athymic nude mice**. A. Mean body weight and NPC tumor burden treated with LY294002. B. Upper: treatment with LY294002(75 mg/kg); Under: no treatment group. C. Representative areas are shown (× 400 magnification). TUNEL-positive cells in orthotopic tumor xenografts (Lift: no treatment group; Right: treatment with 75 mg/kg LY2940020). D. Apoptosis rate induced by different concentrations of LY294002 in tumors xenografts in athymic nude mice.

Tumors generated by orthotopic implantation of the metastatic CNE-2Z cell line were used to evaluate the effect of LY294002 on proliferation and apoptosis in an orthotopic xenograft model. All of the mice were sacrificed after 4 weeks of treatment. Treatment with LY294002 (50 mg/kg, 75 mg/kg) significantly reduced mean NPC tumor burden as compared with the control group (LY294002 50 mg/kg, 75 mg/kg; P < 0.001). Treatment with 10 mg/kg or 25 mg/kg LY294002 was less effective in decreasing tumor burden. Mean NPC tumor burden treated with LY294002 was remarkably decreased in a dose-dependent manner, whereas mean body weight was no obvious difference between control and treated groups (LY294002 10 mg/kg, 25 mg/kg, 50 mg/kg, and 75 mg/kg; *P > 0.05*; Fig 4A and 4B). Compared with control, TUNEL-positive cells treated with LY294002 were significantly increased in a dose-dependent fashion (Fig 4C and 4D), with significant difference (*P < 0.01*).

### Immunohistochemical studies for xenograft tumor tissues

**Figure 5:**
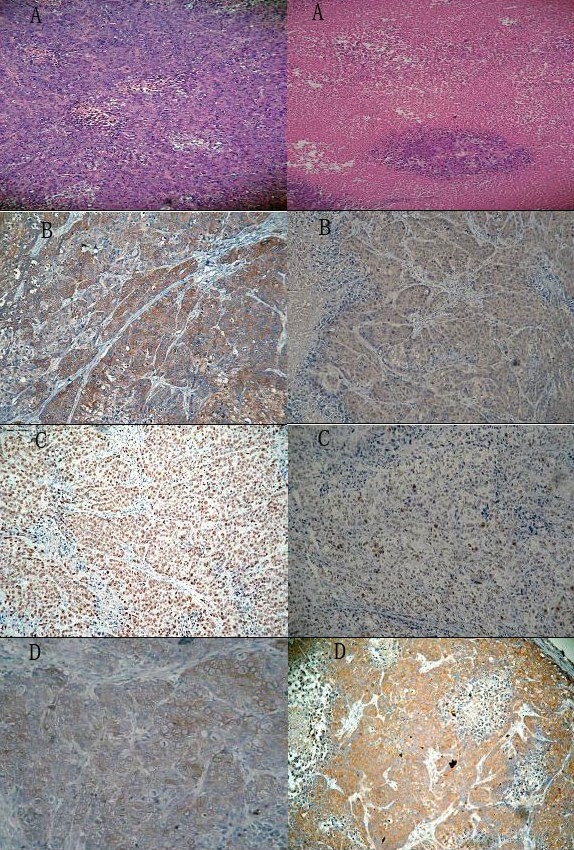
**Histological examination and immunohischemistry analysis of tumors xenografts in athymic nude mice**. A Representative areas are shown (× 200 magnification). Histological appearance of tumor tissue from CNE-2Z-inoculated athymic mice with or without PI3K inhibitor treatment (Left: no treatment group; Right: treatment with 75 mg/kg LY2940020). B Expression of p-Akt in the tumor tissue with or without PI3K inhibitor treatment (Left: no treatment group; Right: treatment with 75 mg/kg LY2940020). C Expression of Ki67 in the tumor tissue with or without PI3K inhibitor treatment (Left: no treatment group; Right: treatment with 75 mg/kg LY2940020). D Expression of caspase-9 in the tumor tissue with or without PI3K inhibitor treatment (Left: no treatment group; Right: treatment with 75 mg/kg LY2940020).

Finally, the histological examination and immunohischemistry were performed to determine the biological influence of LY294002 on tumor morphology, proliferation, apoptosis, and expression of Akt, phosphorylated Akt. The histological changes showed that tumor cells of treated groups were more necrosis than those of control group (Fig 5A). Compared with control group, the expression of phosphorylated Akt was significantly decreased in treated with LY294002 (Fig 5B). Results of immunohistochemical staining with Ki67 and caspase-9 support the gross observations. A great many of NPC cells from the control group stained positive Ki67 (Fig 5C). The number of proliferation cells treated with LY29400 showed significant reduction in a dose-dependent manner (Fig 5D), with significant difference (*P < 0.05; P < 0.01*). The expression of caspase-9 appeared to have an obvious increase in the groups treated with LY294002 (Fig 5E). No significant difference was found between the expression of Akt in tumor from the control and LY294002-treated mice.

## Discussion

The PI3K/Akt cascade is known to be an important survival factor in the signal transduction cascades involved in the cell survival and apoptosis. PI3K is one of the core intracellular signaling molecules in the stimulation of growth factors, subsequently phosphorylating and activating Akt. This signaling pathway cascades activated by some other factors play a critical role in regulating tumor cell growth, survival, motility, invasion, and differentiation. Although there has been a rapid expansion in the number of identified physiological Akt substrates that are involved in various aspects of cellar function, there are clearly candidates that are directly involved in the regulation of apoptosis [2]. Akt can suppress apoptosis by directly interacting with and phosphorylating these proapoptotic proteins. This cascade is thus an exciting new target for molecular targeting therapy for cancer. Our results show that LY294002 markedly inhibited NPC CNE-2Z cell growth, proliferation, and induced apoptosis in vitro and in vivo.

Previous studies have demonstrated that the expression of phosphorylated Akt had a closely correlated to cell growth, proliferation, and resistance to apoptosis [9,15,22-25]. In addition, LY294002, the PI3K/Akt specific inhibitor, showed the growth-inhibitory effects due to cell-cycle arrest closely correlated to with the accumulation of cyclin-dependent kinase inhibitors p27 and PTEN [6,7,26,27]. Some studies found that PI3K inhibitors produce apoptosis and antiproliferative effects on pancreatic carcinoma cells in vivo and in vitro [15,23].

To evaluate the role of Akt in the biology of NPC, we used immunoblotting to analyse the relationship between phosphorylation-specific antibody to demonstrate Akt activity in cultured cells and then confirmed the ability of the LY294002 to decrease Akt phosphorylation in NPC cell line and xenograft tumor tissue. We examined the effect of LY294002 on cell proliferation and the induction of apoptosis. However, there was a great discrepancy between the sensitivity to LY294002 and the level of expression of phosphorylated Akt. The degree of CNE-2Z cell proliferation and apoptosis was shown in a dose-dependent fashion. Western blot results revealed decreasing of phosphorylated Akt levels with increasing dose of LY294002. In tumor sections from athmic mice, the necrotic region treated with a higher dose LY294002 (50 mg/kg and 75 mg/kg) was more great than those of the lower dose (10 mg/kg, 25 mg/kg) of LY294002 and the control group. The mean body weight did not exhibit significant differences between the groups treated with LY294002 and control group. However, compared with LY294002 (10 mg/kg, 25 mg/kg) and control group, the mean tumor burden was remarkably decreased in treated with LY294002 (50 mg/kg, 75 mg/kg) group, with significant difference.

Because the PI3K/Akt signaling pathway plays an important role in many aspects of cellular homeostasis [1,4], it is necessary concern that PI3K inhibitor would interfere with the survival and proliferation of critical populations of normal cells and show unacceptable toxicity. Previous experiments have testified that it was safe biweekly i.p. administration of under to 100 mg/kg of LY294002 [15]. The dose (50 mg/kg and 75 mg/kg) of LY294002 produced obvious inhibition of Akt phosphorylation, reduced tumor cell proliferation, and increased apoptosis in orthotopic CNE-2Z NPC xenografts.

Akt specific inhibitor, LY294002, did not cause obvious apoptosis at 24 h exposure, but induced greatly apoptosis in 48 h in a time-dependent manner. Some studies have clearly shown that Akt phosphorylates and inactivates cspase-9, an important initiation caspase of the mitochondria pathway-mediated apoptosis [21,28]. Our data indicated that the caspase-9 inhibitor ZVAD completely blocked apoptosis induced by PI3K inhibitor, and suggested that AKT conferred resistance to LY294002-induced apoptosis ultimately through suppressing caspase activation pathways in CNE-2Z cells. The results of specific caspase inhibitor demonstrated that blocking caspase-9 pathway exerted a much greater protective effect against apoptosis.

## Conclusion

In summary, Akt played a critical role in regulating the sensitivity of CNE-2Z cells to the induction of apoptosis by LY294002. This kinase pathway conferred resistance by suppressing caspase-9 cascade.

## Competing interests

The authors declare that they have no competing interests.

## Authors' contributions

GZ and HJ designed the experiments, HJ carried out most of experiments and drafted the manuscript. XL and HD assisted with animal experiments. DF participated in statistical analysis and interpretation of data. All authors read and approved the final manuscript.
